# HOXC10 Promotes Metastasis in Colorectal Cancer by Recruiting Myeloid-derived Suppressor Cells

**DOI:** 10.7150/jca.76945

**Published:** 2022-09-06

**Authors:** Jiao Yu, Xiaojiao Chen, Shuhong Zhao, Jingchen Jing, Qing Wang, Yunzhi Dang

**Affiliations:** 1 Department of Radiation Oncology, Shaanxi Provincial People's Hospital, Xi'an, 710086, China; 2 Xi'an Medical University, Xi'an, 710086, China

**Keywords:** colorectal cancer, HOXC10, myeloid-derived suppressor cells.

## Abstract

**Background:** Since metastasis is the primary cause of death in human colorectal cancer (CRC) patients, the exact mechanism underlying CRC metastasis remains unclear. Here, we provide evidence for a unique function of HomeoboxC10 (HOXC10) in driving CRC metastasis, as well as treatment options for these subpopulation patients.

**Methods:** Immunohistochemistry detected the expression of HOXC10 in the human CRC cohort. The function of HOXC10 in CRC metastasis was investigated using the cecum orthotopic model.

**Results:** In CRC patients, elevated expression of HOXC10 expression was linked to lymph node metastases, distant metastasis, worse tumor differentiation, higher AJCC stage, and poor prognosis. HOXC10 is also an independent predictive predictor for CRC patients (P<0.001). HOXC10 overexpression increased the metastasis ability of MC38 cells and promoted the infiltration of MDSCs by upregulating CXCL5 at the same time. The CXCR2 inhibitor can reduce the rate of metastasis in MC38 cells by reducing MDSCs infiltration. SB225002, a CXCR2 inhibitor, and anti-programmed death-ligand 1 (anti-PD-L1) can significantly prevent CRC metastasis.

**Conclusions:** HOXC10 overexpression upregulated CXCL5, which promoted MDSCs infiltration. Interrupting this loop might be a potential therapy option for HOXC10-induced CRC metastasis.

## Introduction

Colorectal cancer (CRC) ranks as the third most commonly diagnosed cancer and fourth most lethal malignancy, accounting for almost 900,000 deaths per year 1. Although advances in treatment methods have improved overall survival rates for CRC patients, the 5-year survival rate is still only 12-14% in patients with metastatic CRC 2. Therefore, further research into the underlying mechanisms of CRC metastasis and the development of more effective therapies remains critical. Although immunotherapy has demonstrated encouraging therapeutic results in a variety of cancers, the response rate is still low because of immunosuppression and immune evasion. Anti-PD-1 and AMG510 produced a long-lasting anti-tumor effect, which indicates that combined therapy may provide a promising treatment option for specific CRC subpopulations 3.

The Homeobox (HOX) family of transcription factors is critical in organ development and can regulates apoptosis, receptor signaling, differentiation, motility, and angiogenesis 4. In the past decades, deregulation of HOX family genes has shown to have a key role in cancer initial and progression 4. In addition, HOX genes that are not normally expressed in a tissue might become overexpressed in malignancies 5. The HOXC10 gene, which belongs to the HOX gene family, has an important role in several physiological processes in mammals, including limb morphological development, limb regeneration, and lumbar motor neuron differentiation 6-8. In malignancies, overexpression of HOXC10 was shown to be associated with bad pathologic stage and poor prognosis 9-10. HOXC10 upregulation can promote tumor proliferation 11, invasion 12, and metastasis 13. Overexpression of HOXC10 in KRAS-mutant lung tumor predicts poor response to combination BET/MEK inhibitors, while its reduction mediates the therapeutic effect 14. In previous study, it was shown that the expression of HOXC10 was significantly higher in tumor tissues compared with the normal tissues. However, the study included only 39 patients 15. Xie Ying, et al found that HOXC10 mRNA and protein expression levels were significantly upregulated in CRC tissues and cells. Mechanically, HOXC10 overexpression activated MTFR2 expression to enhance the proliferation, clone formation, invasion and migration of CRC cells. However, the study did not perform the animal study, which was a limitation of this study 16. In addition, Chen et al, found that cis-HOXC10 is robustly expressed in colorectal tumor-initiating cells (TICs). Mechanistically, cis-HOX binds to HOXC10 mRNA to attenuate its decay and drives colorectal tumorigenesis and stemness. However, the function and mechanism of HOXC10 in CRC metastasis was not further investigated in this study 17. These studies suggested that HOXC10 may play a role in CRC progression. However, the function of HOXC10 in CRC and the underlying molecular mechanism needs further investigation.

Immunosuppressive myeloid cells, such as myeloid-derived suppressor cells (MDSCs), are important mediators in helping tumors escape immune surveillance, which contributes to the advancement of CRC malignancy 5. MDSCs represent a heterogeneous population of myeloid progenitors that accumulate in the tumor microenvironment and serve as potent pro-inflammatory mediators in suppressing T cell functions and contribute to immune evasion 18. Activated MDSCs express a source of secreted cytokines and enzymes, which suppress T cells and natural killer (NK) cells while activating Tregs 19-20. In addition, MDSCs can also boost tumor cell survival, angiogenesis, invasion, and metastasis, and depleting MDSCs can improve anti-PD-L1 effectiveness dramatically 21. In CRC, several chemokines, such as CXCL1, CXCL5, CSF1, CCL2 can promote the development and recruitment of MDSCs 22-25. MDSCs can boost the progression of CRC by suppressing the immune system, controlling bioavailability, and triggering the epithelial to mesenchymal cell transition (EMT). In CRC, the precise oncogenic signal that causes MDSCs recruitment and activation is yet unknown. More critically, the benefit of targeting MDSCs in combination with other treatments for CRC is uncertain.

Here, we found that HOXC10 was upregulated in CRC patients and associated with poor prognosis. HOXC10 overexpression increased CRC metastasis by upregulating CXCL5 expression. CXCR2 inhibitor SB225002 and anti-PD-L1 combined treatment can significantly reduce HOXC10-mediated CRC metastasis.

## Materials and Methods

### Cells and culture

All human cell lines were purchased from The American Type Culture Collection (ATCC), Manassas, VA, USA. The human cell lines SW620 and SW480 were cultured in RPMI-1640 (Gibco) medium with 10% FBS, 100 μg/ml penicillin, and 100μg/ml streptomycin. Murine colon cancer cell line MC38 was purchased from OBiO Technology (Shanghai) Co., Ltd. The MC38 cells was cultured in Dulbecco's Modified Eagle Medium (DMEM, Gibco) medium with 10% FBS, 100μg/ml penicillin, and 100 μg/ml streptomycin.

### Immunohistochemistry

This study was approved by the ethics committee of Shaanxi Provincial People's Hospital, and informed consent was written and based on the ethical guidelines of the 1975 Declaration of Helsinki. Furthermore, human subjects' private rights were always respected. A tissue microarray was created using CRC specimens and matched neighboring tissues (Shanghai Biochip Co, Ltd. Shanghai, China). The tissue microarray was stained for HOXC10 (Abcam, ab153904), CD11b (Abcam, ab6640) and CD8 (Abcam, ab4055) expression. Immunohistochemistry was carried out on 4 μm thick paraffin-embedded slices that had been normally treated. Images were captured using an Olympus light microscope with a DP70 digital camera (Olympus, Japan).

Immunohistochemistry was performed according to standard procedures as outlined in our previous study 26. Briefly, after baking on a panel at 60 °C for an hour, the tissue sections were deparaffinized with xylene and rehydrated through gradient ethanol immersion. Endogenous peroxidase activity was quenched by 3% (vol/vol) hydrogen peroxide in methanol for 12 min, followed by three 3-min washes with phosphate-buffered saline (PBS). Then the slides were immersed in 0.01 mol/L citrate buffer solution (pH=6.0) and placed in a microwave oven for 30 min. After washing in PBS (pH=7.4, 0.01 mol/L), the sections were incubated in a moist chamber at 4 °C overnight with the primary antibody diluted in PBS containing 1% (wt/vol) bovine serum albumin. Negative controls were performed by replacing the primary antibody with preimmune mouse serum. After three 5 min washes with PBS, the sections were treated with a peroxidase-conjugated second antibody (Santa Cruz) for 30 min at room temperature, followed by additional three 5 min washes with PBS. Reaction product was visualized with diaminobenzidine for 2 min. Images were obtained under a light microscope (Olympus, Japan) equipped with a DP70 digital camera.

Analyses were performed by two independent observers who were blinded to the clinical outcome. The immunostaining intensity was scored on a scale of 0 to 3: 0 (negative), 1 (weak), 2 (medium), or 3 (strong). The percentage of positive cells was evaluated on a scale of 0 to 4: 0 (negative), 1 (1%-25%), 2 (26%-50%), 3 (51%-75%), or 4 (76%-100%). The final immuno-activity scores were obtained by multiplying the two scores above, yielding a total score of 0~12. Each case was ultimately considered “negative” if the final score ranges from 0~3 and “positive” if the final score ranges from 4~12.

### Statistical analysis

Statistics were calculated with SPSS software (version 20.0). P values were statistically analyzed by the χ^2^ test for categorical variables and by Student's test for quantitative data. The recurrence and survival data were analyzed by the Kaplan-Meier method. Cox proportional hazards model was used for univariate and multivariate analyses. Differences were considered statistically significant when p < 0.05.

## Results

### Elevated HOXC10 positively correlates with poor prognosis in CRC patients

**Figure 1:**
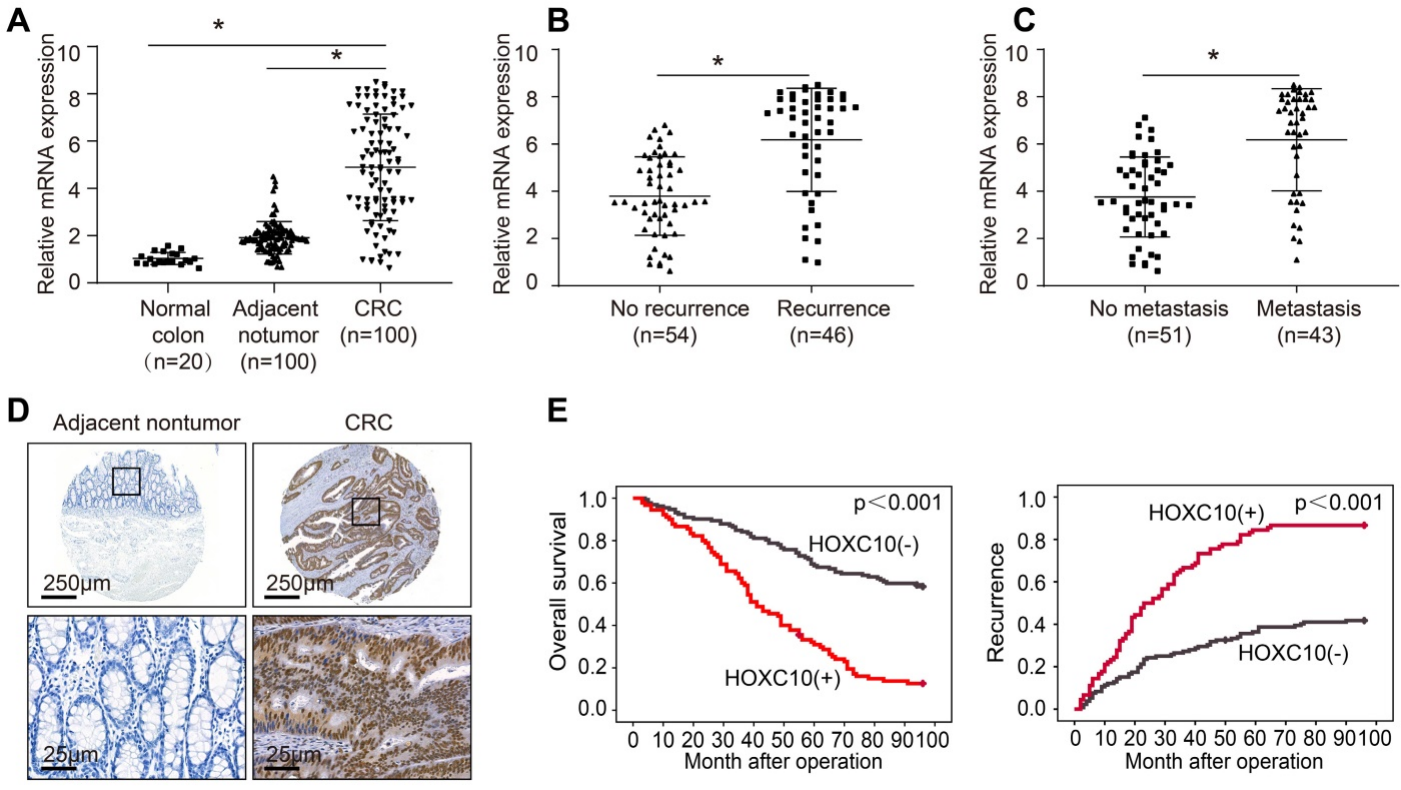
** Elevated HOXC10 positively correlates with poor prognosis in CRC.** (A). Relative HOXC10 mRNA expression in 20 normal colon tissues and 100 paired CRC and adjacent nontumor tissues. (B). Relative HOXC10 mRNA expression in CRC tissues with recurrence or with no recurrence. (C). Relative HOXC10 mRNA expression in CRC tissues with metastasis or with no metastasis. (D). Representative images of IHC staining of HOXC10. (E). Kaplan-Meier analysis of the correlation of HOXC10 expression with overall survival and recurrence in human CRC cohort.

To investigate the function of HOXC10 in CRC, we detected its mRNA expression in 20 colorectal epithelial specimens and 100 paired CRC and adjacent nontumor specimens. The HOXC10 mRNA levels in CRC tissues were greater than in non-tumor tissues and normal colorectal epithelial specimens (Figure 1A). The HOXC10 mRNA level was significantly higher in patients with recurrence than those with no recurrence (Figure 1B). Furthermore, the amount of HOXC10 mRNA in patients with metastasis was greater than in patients without metastasis (Figure 1C).

In order to determine the clinical significance of HOXC10 in CRC patient survival, we used a tissue microarray of 222 CRC samples to profile the expression of HOXC10 in order to identify the clinical importance of HOXC10 in CRC patient survival. The immunohistochemistry pictures revealed that CRC tissues had considerably greater HOXC10 expression than non-tumor tissues (Figure 1D). The elevated HOXC10 expression was positively correlated with tumor differentiation, tumor invasion, lymph node metastasis, distant metastasis, and higher American Joint Committee on Cancer (AJCC) stage (Table 1). Overexpression of HOXC10 was found to be an independent predictor of poor overall survival in multivariate analysis (Table 2). Furthermore, the Kaplan-Meier curve showed that patients with positive HOXC10 expression had reduced overall survival time and higher recurrence rates than those with negative HOXC10 expression (Figure 1E). In addition, all these works indicate that HOXC10 overexpression was found to be a predictive factor in CRC patients.

### Elevated expression of HOXC10 promotes CRC metastasis in immunocompetent mice

**Figure 2:**
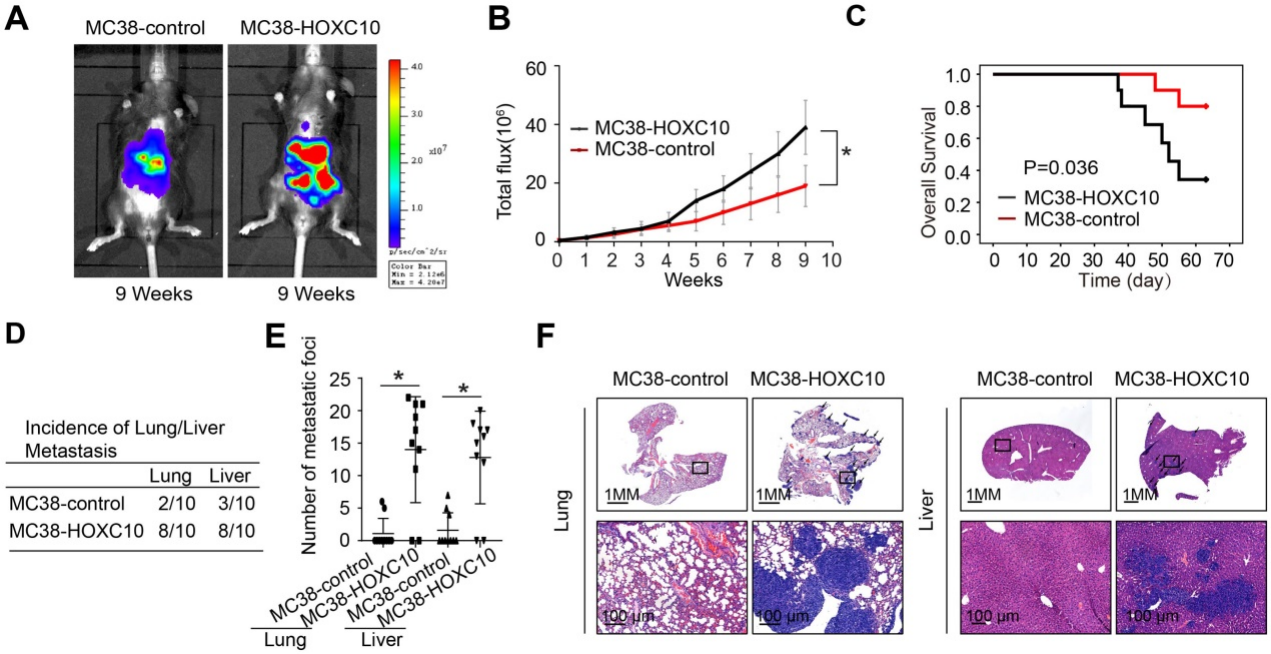
** HOXC10 overexpression promotes CRC metastasis in immunocompetent mice.** (A-F). In vivo assays showed that HOXC10 overexpression can promote CRC metastasis. (A). Bioluminescent images. (B). Bioluminescent signals. (C). Overall survival. (D). The incidence of lung and liver colonization. (E). The number of lung and liver colonization. (F). HE staining was applied to exhibit metastatic lung and liver nodules. * P<0.05.

CRC is usually caused by underlying chronic colon inflammation and a weakened immune system. Thus, we postulated that HOXC10 might promote CRC progression by changing the immune microenvironment. In MC38 cells, we used lentivirus to upregulation of HOXC10. The cecum orthotopic model in C57BL/6 mice was used to investigate the role of HOXC10 in CRC metastasis. Overexpression of HOXC10 was shown to boost the intensity of the bioluminescent imaging signal (Figure 2A-B). Upregulated HOXC10 can enhance the rate of lung and liver metastasis, as well as the number of metastatic nodules, according to histological investigation (Figure 2D-F). Furthermore, HOXC10 has been shown to reduce the overall survival of C57BL/6 mice (Figure 2C). These findings showed that HOXC10 overexpression is essential for CRC metastasis.

### HOXC10 promotes CRC metastasis by recruitment of MDSCs

**Figure 3:**
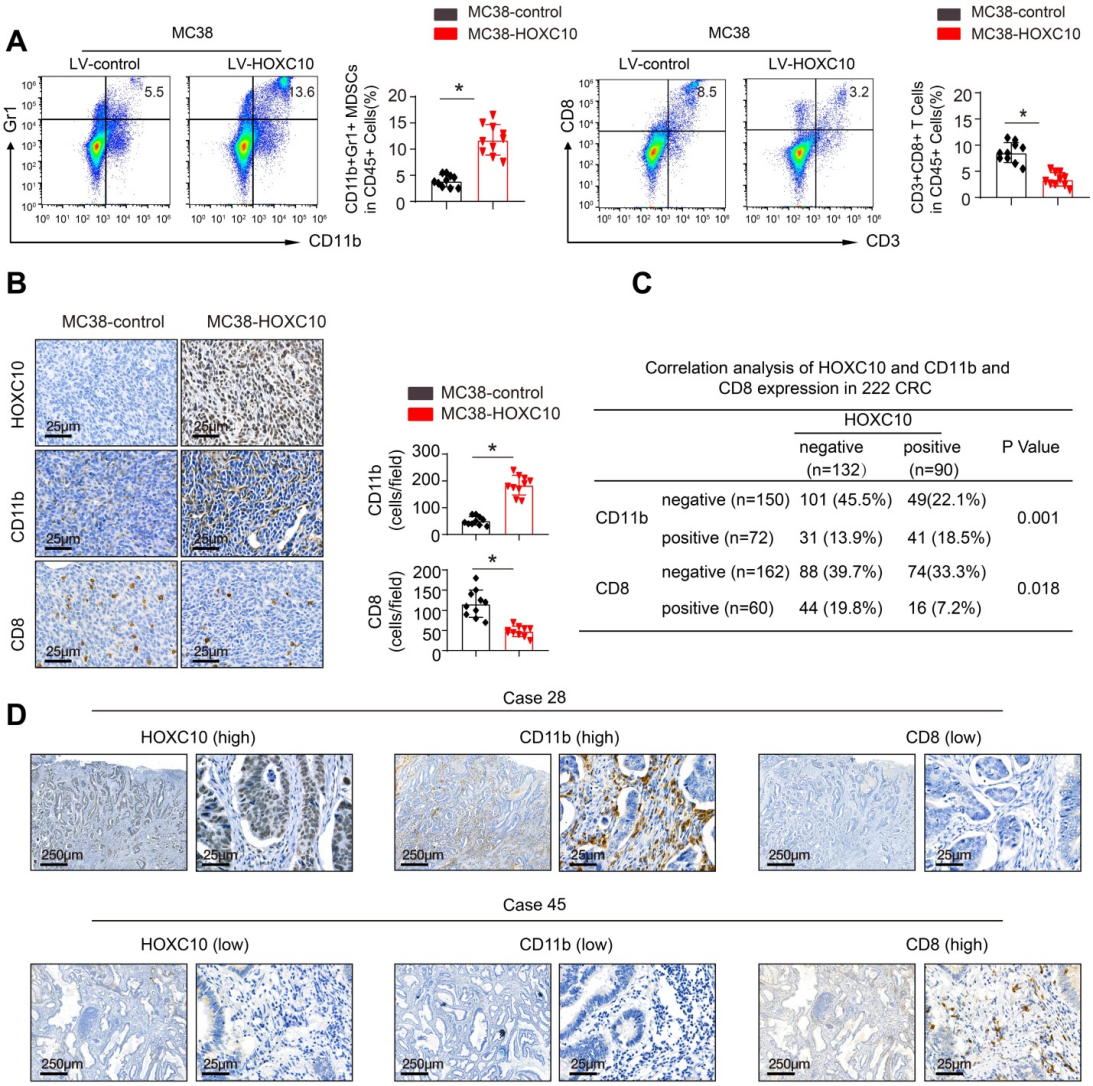
** HOXC10 promotes CRC metastasis by recruitment of MDSCs.** (A). The infiltration of MDSCs and CD8 cells in the MC38-control and MC38-HOXC10 groups was analyzed by flow cytometry. (B). The infiltration of MDSCs and CD8 cells in two group was analyzed by IHC. (C). The correlation between HOXC10 expression and the expression of CD11b or CD8 in CRC cohort. (D). IHC staining showed HOXC10, CD11b and CD8 expression in CRC tissues.

To investigate the mechanisms underlying HOXC10-mediated CRC metastasis, we investigated the cellular immune response. Flow cytometric analysis was used to determine the proportion of immunological and inflammatory cells invading the tumor. In comparison to MC38-control cells, transplantation of MC38-HOXC10 cells dramatically boosted MDSCs infiltration (indicated by CD45+/CD11b/Gr1+), while decreasing CD8+T cell accumulation (defined by CD45+/CD3+/CD8+) (Figure 3A). In addition, IHC staining revealed that MDSCs infiltration was much higher in MC38-HOXC10 tumors, while CD8+ T cell infiltration was lower (Figure 3B). In the CRC cohort, IHC labeling was used to assess HOXC10, intratumoral MDSCs (using CD11b as a marker), and CD8 expression. Figure 3D shows representative IHC pictures of HOXC10, CD11b, and CD8 expression. In the CRC cohort, HOXC10 expression was positively related with CD11b expression but negatively associated with CD8 expression (Figure 3C-D).

### HOXC10 overexpression induces MDSCs chemotaxis by upregulating CXCL5 expression in CRC

To investigate the mechanism through which HOXC10 recruits MDSCs, we used SW480 CRC cells to create SW480-HOXC10 stable cells by lentivirus. Using an Affymetrix PrimeView Human Gene Expression Array, we examined transcriptome alterations in SW480-HOXC10 and SW480-control cells. The upregulation of HOXC10 can increase the expression of various metastasis-related genes, including as CXCL5, CCL17, CCL14, and CXCR2 (Supplementary Table 1). Previous studies indicated that tumor cell-derived CXCL5 could promote CRC metastasis and associated with poor prognosis in CRC patients 22, 27. We hypothesized whether HOXC10 promotes MDSCs infiltration by upregulating CXCL5 expression.

**Figure 4:**
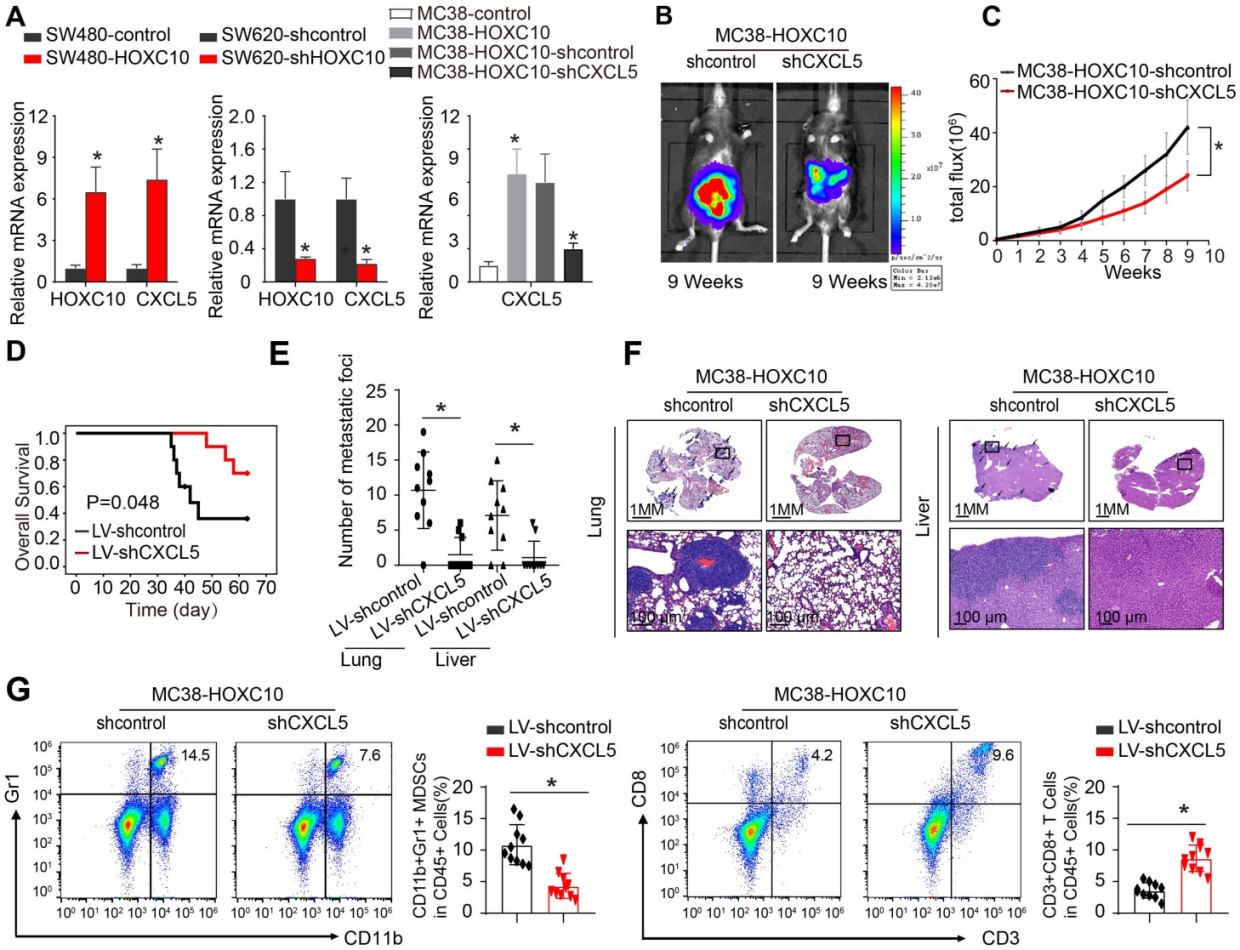
** HOXC10 overexpression induces MDSCs chemotaxis by upregulating CXCL5 expression in CRC.** (A). HOXC10 and CXCL5 expression in the indicated cell by Real-time PCR analysis. (B-F). CXCL5 knockdown can inhibit HOXC10-mediated CRC metastasis. Bioluminescence images (B). Bioluminescence signals (C). Overall survival (D). The number of lung and liver colonization(E). HE staining (F). (G). The infiltration of MDSCs and CD8 in two groups was analyzed by flow cytometry. All the data are shown as the mean±s.d. * P<0.05.

The real-time assay showed that upregulation of HOXC10 can increase the CXCL5 expression in SW480 cells, whereas downregulation of HOXC10 can markedly reduce CXCL5 expression in SW620 cells (Figure 4 A). Then, to examine the role of CXCL5 in CRC metastasis, we used lentivirus to downregulate CXCL5 in MC38-HOXC10 cells and developed the cecum orthotopic tumor implantation model in C57BL/6 mice. CXCL5 knockdown can considerably lower lung and liver metastases rates while also lengthening the overall survival in the MC38-HOXC10 group compared to the control group (Figure 4B-F). Furthermore, IHC staining revealed that knocking down CXCL5 expression in MC38-HOXC10 cells reduces MDSCs infiltration, while it increases CD8+ T cell infiltration when compared to control (Figure 4G). Taken together, the HOXC10-CXCL5 axis plays a critical role in attracting MDSCs to the CRC TME, and inhibiting this axis can drastically reduce HOXC10-mediated CRC metastasis.

### Combined application of CXCR2 inhibitor SB225002 and anti-PD-L1 dramatically blocks HOXC10-mediated CRC metastasis

**Figure 5:**
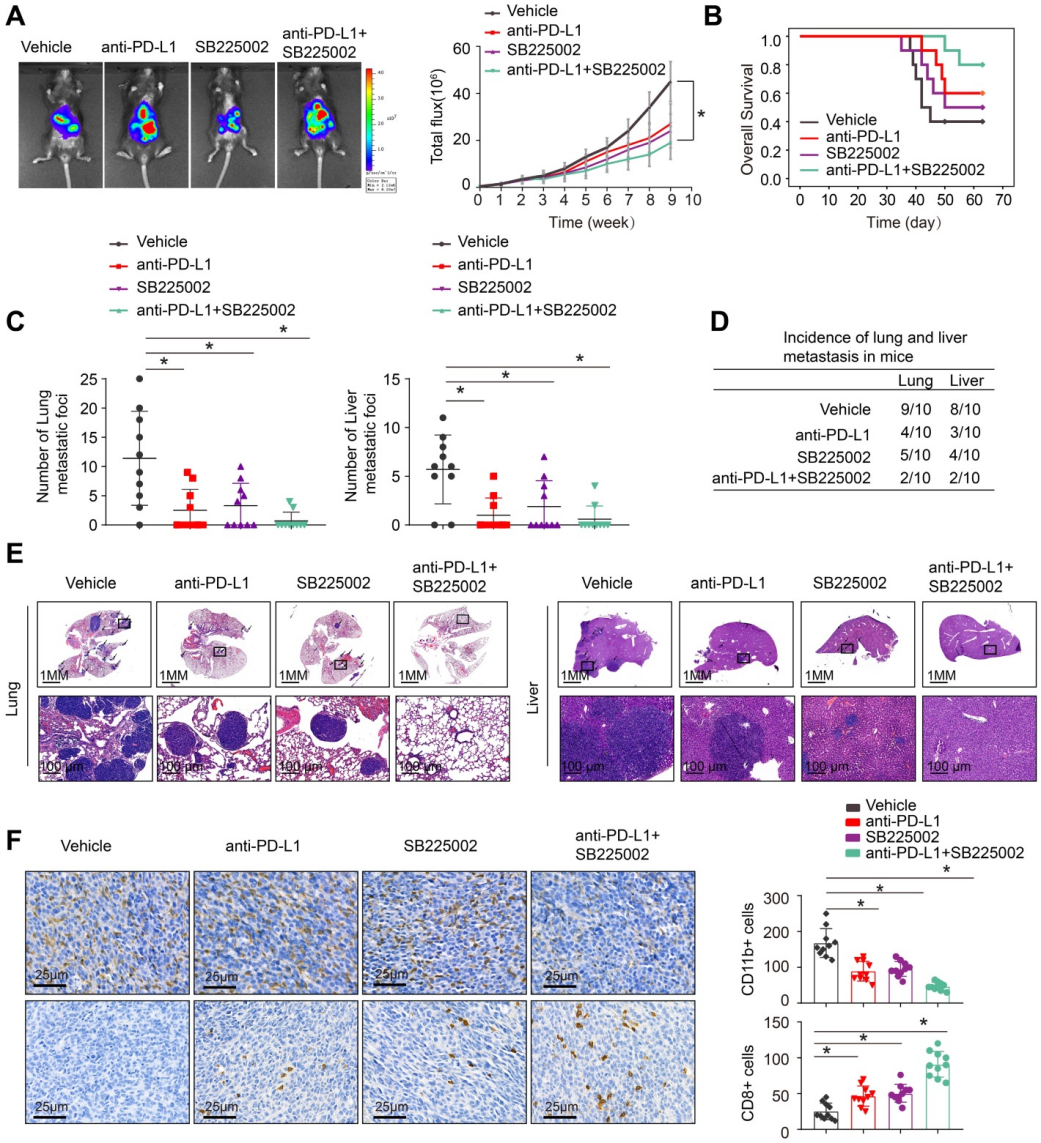
**Combined application of CXCR2 inhibitor SB225002 and anti-PD-L1 dramatically blocks HOXC10-mediated CRC metastasis.** (A-E). One week after injection of MC38-HOXC10 cells, mice in four groups were treated with vehicle, PD-L1 antibody or/and SB225002 (n=10 mice/group) until treatment endpoint. *In vivo* assays showed that combined treatment of PD-L1 antibody and CXCR2 inhibitors SB225002 can almost inhibit CRC metastasis. (A). Representative bioluminescent images and bioluminescent signals in indicated group. (B). Overall survival in indicated group. (C). The number of lung and liver nodules in indicated group. (D). The incidence of lung and liver nodules in indicated group. (E). Representative HE staining of lung and liver tissues. (F). IHC staining detected the infiltration of MDSCs or CD8+ cells in indicated group. All the data are shown as the mean±s.d. * P<0.05.

The depletion of MDSCs showed a synergistic effect with anti-PD-L1 in CRC 21, 22. SB225002, a selective CXCR2 inhibitor, was found to have a potential therapeutic impact, reducing MDSCs infiltration and increasing anti-tumor T cell function via enhancing CD8+ T cell activation 22, 28. SB225002 was tested to see whether it may improve CRC response to anti-PD-L1 blocking, the luciferase-labeled MC38-HOXC10 was injected to cecal wall in C57BL/6 mice under anesthesia (n=10 for each group). The *in vivo* metastatic assay revealed that SB225002 anti-PD-L1 medication alone can reduce the rate of lung and liver metastasis and metastatic nodules, while increased the overall survival time of MC38-HOXC10 group mice. Compared to SB225002 or anti-PD-L1 alone, the combination of SB225002 and anti-PD-L1 dramatically reduced lung and liver metastasis rate and metastatic nodules, with a considerably longer survival time (Figure 5A-E).

To investigate the underlying mechanism of the antitumor response triggered by the combination of SB225002 and anti-PD-L1 antibody, we analyzed the infiltration of MDSCs and CD8+T cells in MC38-HOXC10 orthotopic CRC tumors by IHC. In the combined therapy group, IHC labeling reduced MDSCs infiltration while increasing CD8+ T cell infiltration (Figure 5F). These findings showed that inhibiting PD-L1 and MDSCs together can greatly reduce HOXC10-mediated CRC metastasis.

## Discussion

The molecular mechanisms that confer competence of CRC cells to metastasis and interact with the microenvironment are poorly understand. New evidence suggests that HOXC10 acts as an oncogene and can promote tumor proliferation 11, invasion 12 and metastasis 13. In this study, we found that HOXC10 was shown to be a poor predictor of CRC patients. HOXC10 overexpression was linked to lymph node metastases, distant metastasis, worse tumor differentiation, and higher TNM stage. Furthermore, in immune-competent animals, upregulation of HOXC10 can promote CRC metastasis by recruitment MDSCs. These findings indicate that HOXC10 overexpression is essential for CRC metastasis. Compared with previous study 15-17, we systematically revealed the function and the underlying mechanism of HOXC10 in CRC metastasis, and may provide a promising treatment strategy for HOXC10 overexpression CRC subpopulation.

Suppression of antitumor immune responses is one of the major hallmarks of cancers. MDSCs represent the main immunosuppressive cells present in the TME that sustain cancer progression 29. In CRC, MDSCs are widely considered the link between chronic inflammation and cancers 5. Thus, a deeper understanding of the molecular mechanism recruitment of MDSCs in CRC may be beneficial to the development of novel treatment methods for CRC patients. In this study, we demonstrated that HOXC10 overexpression causes immunological escape in CRC via MDSCs chemotaxis. CXCL5, a ligand for CXCR2, involved in immune cell recruitment and promotes angiogenesis, tumor development, and metastasis 30. The CXCL5-CXCR2 axis enhances CRC spread by stimulating tumor angiogenesis, enhancing the epithelial-mesenchymal transition (EMT), and upregulating PD-L1 expression, according to many recent studies 25, 31. In this work, we discovered that the chemokine CXCL5 is involved in the recruitment of MDSCs, which promotes tumor metastasis. Furthermore, inhibiting the CXCL5-CXCR2 axis by CXCR2 inhibitor can result in reduce MDSCs infiltration and show therapeutic effects.

Mounting evidences showed that molecular targeted therapies in cancers targeting subpopulation with a molecular alteration shows promising response 32. In particular, the combination of anti-PD-1/anti-PD-L1 with anti-CTLA4 agents, locoregional therapies or VEGF/VEGFR inhibitors synergistically enhances antitumor immunity 33-35. Then, we set out to take a pharmacological approach to break up the positive feedback loop reported in our study, and we focused on the CXCR2 inhibitor. SB225002, a selective CXCR2 inhibitor, was found to have a potential therapeutic impact, reducing MDSCs infiltration and increasing anti-tumor T cell function 22, 26. This study hypothesized that that combining PD-L1 antibody and CXCR2 inhibitors can result in synergistic effectiveness in the CRC subgroup with HOXC10 overexpression. *In vivo* study showed that combining SB225002 with anti-PD-L1 therapy can significantly reduce HOXC10-mediated CRC metastasis when compared to control or single drug. In addition, the combination therapy dramatically reduced MDSCs recruitment while increasing CD8+ T cell infiltration. As a result, inhibiting HOXC10-induced CRC metastases by targeting this positive feedback loop might be a potential combination therapeutic approach.

In summary, we discovered that overexpression of HOXC10 contributed to CRC metastasis by upregulating CXCL5 expression and increased the infiltration of MDSCs into the tumor microenvironment. CXCR2 inhibitor, SB225002 and anti-PD-L1 together can effectively inhibit HOXC10-mediated CRC metastasis. Our findings demonstrated a new mechanism of HOXC10 deregulation and a possible therapy for HOXC10-mediated CRC metastasis.

## Supplementary Material

Supplementary materials and methods, figure and table.

## Figures and Tables

**Table 1:** Correlation between HOXC10 expression and clinicopathological characteristics in cohort of human CRC.

Clinicopathological variables		Tumor HOXC10 expression		*P* Value
		Negative (n=132)	Positive (n=90)	
Age	≤50	59	45	0.437
	>50	73	45	
Gender	female	61	39	0.672
	male	71	51	
Tumor size	≤5cm	62	35	0.233
	>5cm	70	55	
Tumor differentiation	well or moderate	104	52	0.001
	poor	28	38	
Tumor invasion	T1-T3	111	65	0.032
	T4	21	25	
Lymph node metastasis	absent	94	30	<0.001
	present	38	60	
Distant metastasis	absent	119	64	<0.001
	present	13	26	
AJCC stage	I-II	95	27	<0.001
	III-IV	37	63	

**Table 2:** Univariate and multivariate analysis of factors associated with survival and recurrence in cohort of human CRC

Clinical Variables	Time to Recurrence		Overall Survival	
	HR (95% CI)	P value	HR (95% CI)	P value
**Univariate analysis**				
Age (≤50vs > 50)	1.068 (0.759-1.501)	0.707	0.996 (0.980-1.013)	0.646
Gender (female vs male)	0.921 (0.631-1.238)	0.470	0.908 (0.645-1.276)	0.577
Tumor size (≤5 cm vs >5 cm)	0.767 (0.542-1.086)	0.130	0.735(0.519-1.041)	0.083
Tumor differentiation (well/moderate vs poor)	0.171 (0.121-0.230)	<0.001	0.165 (0.109-0.236)	<0.001
Tumor invasion (T1-3 vs T4)	0.32 (0.21-0.432)	<0.001	0.290 (0.210-0.431)	<0.001
Lymph node metastasis (absent vs present)	0.098 (0.053-0.156)	<0.001	0.086 (0.074-0.149)	<0.001
Distant metastasis (absent vs present)	0.088 (0.063-0.143)	<0.001	0.121 (0.075-0.145)	<0.001
AJCC stage (I-II vs III)	0.096 (0.057-0.131)	<0.001	0.079(0.061-0.132)	<0.001
HOXC10 expression (negative vs positive)	0.291 (0.205-0.415)	<0.001	0.283 (0.199-0.403)	<0.001
**Multivariate analysis**				
Tumor differentiation (well/moderate vs poor)	0.800 (0.497-1.286)	0.356	0.762 (0.474-1.225)	0.262
Tumor invasion (IT1-3 vs T4)	0.739 (0.485-1.127)	0.160	0.803 (0.527-1.222)	0.306
Lymph node metastasis (absent vs present)	0.488 (0.155-1.533)	0.219	0.515 (0.198-1.339)	0.174
Distant metastasis (absent vs present)	0.357 (0.209-0.609)	<0.001	0.360 (0.215-0.603)	<0.001
AJCC stage (I-II vs III)	0.264 (0.080-0.837)	0.029	0.237 (0.086-0.648)	0.005
HOXC10 expression (negative vs positive)	0.561 (0.379-0.831)	0.004	0.601(0.407-0.887)	0.010

## Abbreviations

CRC: Colorectal cancer
HOXC10: homeobox C10
HE: hematoxylin and eosin
IHC: immunohistochemistry
BLI: bioluminescent imaging
CXCL5: chemokine (C-X-C motif) ligand 5
CXCR2: chemokine (C-X-C motif) receptor 2
