# Supplementary material for: HOXC10 Promotes Metastasis in Colorectal Cancer by Recruiting Myeloid-derived Suppressor Cells
Source: J Cancer. 2022 Sep 6;13(12):3308–17. doi: 10.7150/jca.76945 (PMC9516010; doi:10.7150/jca.76945)
Supplement: Supplementary file 1 — Supplementary materials and methods, figure and table. [file jcav13p3308s1.pdf]

HOXC10 Promotes Metastasis in Colorectal Cancer by Recruiting Myeloid-derived Suppressor Cells

Jiao Yu[1], Xiaojiao Chen[2], Shuhong Zhao[1], Jingchen Jing[1], Qing Wang[1], Yunzhi Dang[1] ✉

1. Department of Radiation Oncology, Shaanxi Provincial People's Hospital, Xi'an, 710086, China

2. Xi'an Medical University, Xi'an, 710086, China

Corresponding author: Yunzhi Dang, Department of Radiation Oncology, Shaanxi Provincial People's Hospital, Xi'an 710086, China.

E-mail: dangyunzhi@xiyi.edu.cn

Supplementary Material and Methods

Patients

The CRC cohort included 222 patients with CRC who underwent surgery at Xijing hospital between 2006-2008. The inclusion criteria are as follows : a). Participants must be 18 years or older; b). Histologic diagnosis of colorectal adenocarcinoma; c). Participants must receive curative CRC resection; d). Complete clinical-pathologic and follow-up data, and the follow-up time was more than 8 years; e). No pre-operative anti-cancer treatment; f). During follow-up, diagnosis of recurrence and distant metastasis was made by imaging methods, including colonoscopy, ultrasound, CT, MRI, PET, and biopsy if necessary.

The exclusion criteria are as follows: a). The patients received preoperative radiotherapy or chemotherapy; b). Pregnant or lactating females; c). Active or prior secondary malignancy or died of something unrelated to the tumor; d). Objection to the medical study.

The pTNM classification for CRC was based on The American Joint Committee on Cancer/International Union Against Cancer stage system. In addition, 20 cases of normal colonic epithelium and 100 pairs of frozen fresh CRC tissues and peripheral nontumor tissues were collected after surgical resection and stored in liquid nitrogen. These tissue pairs were used to detect the mRNA expression of HOXC10.

Construction of lentivirus and stable cell lines

Construction of lentivirus and stable cell lines Lentiviral vectors encoding shRNAs were generated using PLKO.1-TRC (Addgene) and designated as LV-shHOXC10 (human), and LV-shcontrol. “LV-shcontrol” is a non-target shRNA control. The vector “pLKO.1-puro non-Target shRNA Control Plasmid DNA” (purchased from Sigma, SHC016) contains an shRNA insert that does not target any known genes from any species. Short hairpin RNAs (shRNAs) sequences were: shHOXC10(human), 5'-CCGGCTGGAGATTAGCAAGACCATTCTCGAGAATGGTCTTGCTAATC TCCAGTTTTTG-3'.

Lentiviral vectors encoding the mice HOXC10 genes were constructed in FUW-teto (Addgene) and designated as LV-HOXC10. An empty vector was used as the negative control and was designated as LV-control. Concentrated lentivirus was transfected into the CRC cells with a multiplicity of infection (MOI) ranging from 30 to 50 in the

presence of polybrene (6 μg/ml). Seventy-two hours after infection, CRC cells were selected for 2 weeks using 2.5 μg/ml puromycin (OriGene). Selected pools of knockdown and overexpression cells were used for the follow experiments.

In vitro **invasion and migration assay**

For the migration and invasion assay, a 24 well chamber with 8 μm pore filter (Corning corporation, USA) was used. For migration assay, 5×10^5 cells were seeded into the upper chamber in serum-free medium. For invasion assay, 5×10^5 cells were implanted in the top chamber with Matrigel (Corning corporation, USA). After 24～48 hours, the cells were fixed with 95% ethanol and stained with crystal violet. The mean of triplicate assays for each experimental condition was used.

Real-time PCR

Total RNA was extracted using TRIzol Reagent (Invitrogen), and reverse transcription was performed using the Advantage for RT-PCR Kit (Takara) according to the manufacturer's instructions. For the real-time PCR analysis, aliquots of double-stranded cDNA were amplified using a SYBR Green PCR Kit (Applied Biosystems). For the clinical tissue samples, the fold change of the target gene was determined by the following equation: $2^{-\Delta\Delta Ct}$ ($\Delta\Delta Ct = \Delta Ct^{tumor} - \Delta Ct^{nontumor}$). This value was normalized to the average fold change in the normal colon tissues, which was defined as 1.0. All reactions were performed in duplicate.

In vivo metastatic model and bioluminescent imaging

Our implantation tests were under the approval of the ethics committee of Shaanxi Provincial People's Hospital. All efforts were made to minimize the animals' suffering during the experiments. C57BL/6 mice (5 weeks old) were housed under standard conditions and cared for according to the institutional guidelines for animal care. A metastatic colorectal cancer model in mice was established according to the existing protocol. Luciferase labeled mouse CRC cells (4.0×10^6) were injected into the cecal wall in mice under anesthesia (n=10 for each group). Briefly, the caecum was gently exteriorized and was placed on a scalpel holder, flattened, and stabilized with forceps. This maneuver is crucial to prevent leakage of tumor cells into the caecal lumen or peritoneal cavity. A volume of 100μl (4.0×10^6) cells was injected into the caecal wall. Then, the caecum was returned to the peritoneal cavity, peritoneum and skin were closed by running sutures and wound clips.

Luciferase lentivirus was purchased from Shanghai Genechem Co., Ltd. Concentrated luciferase lentivirus was transfected into the CRC cells with a multiplicity of infection (MOI=50) in the presence of polybrene (6 μg/ml). Seventy-two hours after infection, CRC cells were selected for 2 weeks using 2.5 μg/ml puromycin (OriGene). Then we tested the luciferase infection efficiency. In a 96-well plate, we set up 4 gradient dilution cells (each hole is spaced at a certain distance to prevent mutual interference). Then, 5 μl D-luciferin was added to each hole, and the signal value of each well was measured by a multifunctional enzyme marker. If the cell density were positively

correlated with the signal value, indicated luciferase transfection success.

The in vivo tumor formation and metastases were imaged by bioluminescence. D-luciferin (Xenogen, Hopkinton, MA) at 100 mg/kg was injected intraperitoneally into the mice, and bioluminescence was detected using an IVIS 100 Imaging System (Xenogen). After acquiring photographic images of each mouse, luminescent photos were captured using various (1～60 seconds) exposure times. The resulting grayscale photographic and pseudocolored luminescent images were automatically superimposed using the IVIS Living Image (Xenogen) software. This superimposition was performed to facilitate the matching of the observed luciferase signal with its location on the mouse. The survival of the mice was recorded. At the 9 weeks, the mice were sacrificed by injecting excessive pentobarbital sodium for anesthesia (100 mg/kg, Merck, Germany), and the livers and lungs were collected and underwent histological examination.

Preparation of Single Cell Suspensions

Mice were perfused with PBS and anesthetized, and tumors were dissected using a clean razor. Then, the tumor tissues were digested with DNase I (20 mg/mL, Sigma-Aldrich) and collagenase IV (1.5 mg/mL, Sigma-Aldrich) and placed on a table concentrator, 37°C, for one hour. At the end of one hour, we filtered the dissociated cells through 70 μm pore filters rinsed with fresh media. The 1× red cell lysis was added to the tissues and incubated for 5 minutes to lysis the red blood cell, followed

by another rinse.

Flow Cytometric Analysis

Cells were incubated with anti-mouse CD16/CD32 purified antibody (#101302, clone 93, Biolegend) for 10 minutes to block nonspecific antibodies. Then, the cells were stained with fluorophore-conjugated antibodies. Matched isotype antibodies were used as control. Antibodies against CD11b (FITC, #101205), CD45 (PE/Cy7, #103113), Ly-6G/Ly-6C (Gr-1) (PE, #108407), CD3 (FITC, #100203), CD8 (PE, #100707), were purchased from biolegend. Data were analyzed by Flowjo_V10 software (TreeStar, Ashland, OR).

New Supplementary Figure 1

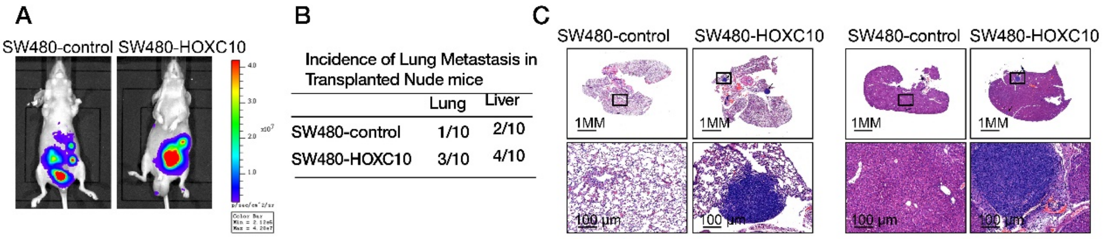

Supplementary Figure 1. (A-C) Metastasis assays of SW480-control and SW480-HOXC10 metastasis ability in the nude mice. (A). Bioluminescent imaging. (B). Representative HE staining of lung and liver tissues. (C). The incidence of lung and liver metastasis.

Supplementary Table 1. Chemokines and Receptors RT2 Profiler PCR Array of SW480-HOXC10 vs SW480-control

Gene	Description	Fold change
CXCL5	chemokine (C-X-C motif) ligand 5	5.51
CCL17	chemokine (C-C motif) ligand 17	4.68

CCL14	chemokine (C-C motif) ligand 14	4.54
CXCR2	chemokine (CXC Motif) receptor 2	4.51
CX3CR1	chemokine (CX3C Motif) receptor 1	4.21
CCL2	chemokine (C-C motif) ligand 2	4.11
CSF1R	colony stimulating factor 1 receptor	4.05
ACKR1	atypical Chemokine Receptors 1	3.85
CCL3	chemokine (C-C motif) ligand 3	3.65
CXCL1	chemokine (C-X-C motif) ligand 1	3.45
CXCL2	chemokine (C-X-C motif) ligand 2	3.21
CCL5	chemokine (C-C motif) ligand 5	2.91
ACKR2	atypical Chemokine Receptors 2	2.85
CXCL6	chemokine (C-X-C motif) ligand 6	2.75
CCL20	chemokine (C-C motif) ligand 20	2.62
CCR2	chemokine (C-C motif) receptor 2	2.59
CXCR1	chemokine (CXC Motif) receptor 1	2.47
CXCL10	chemokine (C-X-C motif) ligand 10	2.43
IL1B	Interleukin-1B	2.39
IL10	Interleukin10	2.35
CCL7	chemokine (C-C motif) ligand 7	2.32
CXCR3	chemokine (CXC Motif) receptor 3	2.30
CCR3	chemokine (C-C motif) receptor 3	2.24
IL4	Interleukin 4	2.23
CXCL11	chemokine (C-X-C motif) ligand 11	2.19
CCL15	chemokine (C-C motif) ligand 15	2.16
CXCL2	chemokine (C-X-C motif) ligand 2	2.11
CX3CL1	chemokine (CX3C Motif) ligand 1	1.98
IL16	Interleukin 16	1.97
SLC7A11	solute carrier family 7-member 11	1.85
IL8	Interleukin 8	1.84
CCL11	chemokine (C-C motif) ligand 11	1.75
CCL7	chemokine (C-C motif) ligand 7	1.69
CXCL11	chemokine (C-X-C motif) ligand 11	1.64
ACKR4	atypical Chemokine Receptors 4	1.59
IL17A	Interleukin 17A	1.55
C5	complement component 5	1.53
SPP1	secreted phosphoprotein 1	1.48
CXCL12	chemokine (C-X-C motif) ligand 12	1.43
CCL22	chemokine (C-C motif) ligand 22	1.40
IL4	Interleukin 4	1.37
AKR1B10	aldo-keto reductase family 1, member B10 (aldose reductase)	1.34
CCL16	chemokine (C-C motif) ligand 16	1.32
CXCL3	chemokine (C-X-C motif) ligand 3	1.29
IL16	Interleukin 16	1.28
CCL4	chemokine (C-C motif) ligand 4	1.26
CXCL13	chemokine (C-X-C motif) ligand 3	1.25
CCR6	chemokine (C-C motif) receptor 6	1.21
CCR9	chemokine (C-C motif) receptor 9	1.15
CXCL14	chemokine (C-X-C motif) ligand 14	1.01
IL8	Interleukin 8	-1.03

CCL18	chemokine (C-C motif) ligand 18	-1.23
CXCR6	chemokine (CXC Motif) receptor 6	-1.31
CXCL9	chemokine (C-X-C motif) ligand 9	-1.35
IL16	Interleukin 16	-1.41
TNF	tumor necrosis factor	-1.52
CCL1	chemokine (C-C motif) ligand 1	-1.57
CCL27	chemokine (C-C motif) ligand 27	-1.61
TLR4	Toll-like receptor 4	-1.65
CCR10	chemokine (C-C motif) receptor 10	-1.71
CXCL16	chemokine (C-X-C motif) ligand 16	-1.75
CCL20	chemokine (C-C motif) ligand 20	-1.81
ACKR3	atypical Chemokine Receptors 3	-1.86
CCL23	chemokine (C-C motif) ligand 23	-1.90
CCR9	chemokine (C-C motif) receptor 9	-1.95
CCL8	chemokine (C-C motif) ligand 8	-2.08
CCR1	chemokine (C-C motif) receptor 1	-2.30
